# Linking ion channel gene expression to neuronal firing patterns through a statistical-biophysical model

**DOI:** 10.1016/j.patter.2025.101390

**Published:** 2025-10-10

**Authors:** Wanjing Huang, Qiang Xu, Sheng Liu

**Affiliations:** 1 State Key Laboratory of Ophthalmology, Zhongshan Ophthalmic Center, Sun Yat-Sen University, Guangdong Provincial Key Laboratory of Ophthalmology and Visual Science, Guangzhou 510060, China

## Abstract

Patch-seq enables the integration of electrophysiological recordings, single-cell RNA sequencing (scRNA-seq), and morphological reconstruction within the same neuron, but establishing mechanistic links between transcriptomic and physiological properties remains a major challenge. Bernaerts et al.^1^ developed a new statistical-biophysical model based on biophysical simulations and modern machine learning techniques. They applied this model to gene expression and established a quantitative link between gene expression and electrophysiological activity patterns. This work is an important advance toward closing the gap between gene expression and neuronal physiology.

## Teaser

Patch-seq enables the integration of electrophysiological recordings, single-cell RNA sequencing (scRNA-seq), and morphological reconstruction within the same neuron, but establishing mechanistic links between transcriptomic and physiological properties remains a major challenge. Bernaerts et al. developed a new statistical-biophysical model based on biophysical simulations and modern machine learning techniques. They applied this model to gene expression and established a quantitative link between gene expression and electrophysiological activity patterns. This work is an important advance toward closing the gap between gene expression and neuronal physiology.

## Main text

Neuronal cell types constitute the fundamental units of the nervous system. Traditionally, neuronal cell types have been distinguished based on their morphological and electrophysiological features. Advances in single-cell transcriptomics have more recently enabled a genetically grounded and increasingly detailed classification of cortical cell types.^2^ However, the extent to which gene expression profiles of individual cell types explain their physiological and anatomical features is still not well understood.

To address this gap, Patch-seq has been developed, combining electrophysiological recordings, single-cell RNA sequencing (scRNA-seq), and morphological reconstruction from the same neuron.^3^^,^^4^^,^^5^^,^^6^ This multimodal approach allows for direct investigation of how a neuron’s gene expression relates to its functional and structural properties. Using this method, previous studies have shown that major neuronal families (e.g., *Pvalb* or *Sst* interneurons or intratelencephalic pyramidal neurons) exhibit characteristic physiological and anatomical traits. Within each family, these properties often vary along a continuum, likely reflecting gradual changes in gene expression.^7^

Another substantial challenge stems from elucidating mechanistic links between neuronal transcriptomic profiles and their electrophysiological characteristics. Although methods such as sparse reduced-rank regression (sRRR) can reveal statistical associations between ion channel gene expression and specific expert-defined electrophysiological features, they do not provide a framework for inferring causality. Motivated by the goal of establishing a quantitative relationship between gene expression and electrophysiological activity, Bernaerts et al. built conductance-based models of neuronal activity with interpretable parameters, fitting them to recordings from hundreds of neurons.^1^ These models then served as a bridge to connect gene expression and electrophysiological traits through sparse regression analysis.

Bernaerts et al. began by analyzing 955 mouse motor cortex (MOp) neurons for which both transcriptomic and electrophysiological data were available.^1^^,^^7^ The “minimal” Hodgkin-Huxley (HH)-based model is a previously established model that effectively represents electrophysiological properties across diverse neuronal families. The authors extended this model by adding key ion channels, resulting in a 13-parameter framework whose simulations were compared to experimental recordings summarized by 23 electrophysiological features. After screening 15 million simulations, the authors identified the best-fitting parameter sets that closely reproduced experimental recordings. However, this approach relied on a large library of precomputed simulations and produced only the single best-fitting parameter sets, without providing any measure of uncertainty.

To resolve this, the researchers applied neural posterior estimation (NPE) with a masked autoregressive normalizing flow to learn the posterior distribution of model parameters and captured the uncertainty of these parameters. They trained the estimator on 7 million simulations of the HH-based model to infer 13 free parameters from 23 electrophysiological features. Once trained, the model could evaluate any experimental recording and return the corresponding posterior distribution without further simulations or training. However, standard NPE failed: posterior samples often produced poor fits or undefined features due to a systematic mismatch between experimental recordings and model simulations. The researchers introduced an additional *r*_*SS*_ (rate to steady state) parameter to improve fits, but mismatches persisted, leading to unreliable posterior inference. To address these, they modified NPE by adding Gaussian noise to features of simulations near experimental data, creating the neural posterior estimation with noise (NPE-N) method. NPE-N significantly improved fit quality, reducing feature-space distance between model and data and ultimately producing more reliable posterior estimates across 955 MOp neurons. Feature-importance analysis revealed that the mean resting membrane potential was the strongest constraint on posterior inference, followed by stimulus potential, spike amplitude, spike threshold, and membrane potential variance.

The paper then investigated how transcriptomic identity relates to electrophysiological properties and the best-fitting HH-model parameters in 955 MOp neurons. NPE-N posteriors varied across cell types: *Pvalb* neurons showed lower uncertainty due to stereotypical firing, while *Vip* neurons showed higher uncertainty due to greater variability. Electrophysiological features from simulated maximum a posteriori traces closely matched experimental data, with pyramidal neurons displaying broader and higher spikes but lower firing rates. Some discrepancies remained, such as higher simulated response latency in interneurons compared with experiments. Mapping HH-model parameters revealed family-specific trends—e.g., high ${\bar{g}}_{Kd}$ (maximal conductance of the delayed rectifier K+ current) in *Pvalb* neurons supporting fast spiking and larger *τ* (time for the membrane potential to increase by 63%, from its resting membrane state during the application of the positive 300 pA current pulse)—with *r*_*SS*_ in pyramidal neurons accounting for broad spikes and delayed responses.

To further advance this work, the researchers aimed to quantitatively investigate how neuronal transcriptomic identity relates to its biophysical parameters. They trained linear sRRR and nonlinear sparse bottleneck neural network models to predict 13 HH-model parameters from gene expression data (focusing on only 427 ion channel genes and marker genes), finding that parameters like delayed rectifying potassium channel ${\bar{g}}_{KV31}$, ${\bar{g}}_{Kd}$, and membrane capacitance *C* were well predicted, while others such as leak potential *E*_leak_ and muscarinic potassium channel conductance ${\bar{g}}_{M}$ were less accurate. At the same time, the *r*_*SS*_ parameter was predicted best. Also, the sRRR model revealed interpretable, mechanistically plausible gene-parameter links, such as *Kcnc1* with potassium channel conductance ${\bar{g}}_{KV31}$ and *Cacna2d1* with calcium channel conductance ${\bar{g}}_{L}$. Predictions were accurate at the family level but weaker at the cell-type level, as shown by lower Euclidean distances between predicted and fitted parameters across families.

In conclusion, this work directly linked the gene expression profiles of a set of cortical neurons to the HH-based model parameters fitted to their electrophysiological signatures. Compared with previous works, which only correlated gene expression with electrophysiology, this represented an important step toward a mechanistic understanding of how a neuron’s gene expression determines its electrophysiological properties.^7^^,^^8^^,^^9^^,^^10^ Bernaerts et al. achieved this by delegating the connection between these quantities and the expression of specific genes to a statistical model.^1^ They first implemented the HH-based model and increased the flexibility by adding ion channel parameters. Then, they modified NPE by adding Gaussian noise and created NPE-N, which outperformed NPE both qualitatively and quantitatively. In the end, their predictive statistical-biophysical model successfully reflected parameter distinctions between major neuron families, yet it struggled with finer cell-type resolution, probably because of dataset limitations, continuous heterogeneity, and noise in transcriptomic data. The authors of the paper hypothesized that fully mechanistic models linking gene expression to electrophysiology would require explicit modeling of mRNA-to-protein translation, a highly complex and still unresolved process. As the authors suggested, combining machine learning with mechanistic models offers a promising path to bridge this causality gap and improve understanding of neuronal diversity.

## Acknowledgments

This work was supported by the National Key R&D Program of China (2022YEF0203200), the National Natural Science Foundation of China (82425016, 82371095, and 32400960) and the Guangdong Basic and Applied Basic Research Foundation (2022A1515110915 and 2024A1515010554).

## Declaration of interests

The authors declare no competing interests.
